# Stochastic Schrödinger equation derivation of non-Markovian two-time correlation functions

**DOI:** 10.1038/s41598-021-91216-0

**Published:** 2021-06-04

**Authors:** Rafael Carballeira, David Dolgitzer, Peng Zhao, Debing Zeng, Yusui Chen

**Affiliations:** 1 Department of Applied Science and Technology, Saint Peter’s University, Jersey City, NJ 07306 USA; 2 Department of Physics, New York Institute of Technology, Old Westbury, NY 11568 USA; 3 Department of Electrical and Computer Engineering, New York Institute of Technology, Old Westbury, NY 11568 USA

**Keywords:** Quantum information, Atomic and molecular physics, Quantum physics, Statistical physics, thermodynamics and nonlinear dynamics

## Abstract

We derive the evolution equations for two-time correlation functions of a generalized non-Markovian open quantum system based on a modified stochastic Schrödinger equation approach. We find that the two-time reduced propagator, an object that used to be characterized by two independent stochastic processes in the Hilbert space of the system, can be simplified and obtained by taking ensemble average over one single noise. This discovery can save the cost of computation, and accelerate the converging process when taking the average over noisy trajectories. As a result, our method can be widely applied to many open quantum models, especially large-scale systems and extend the quantum regression theory to the non-Markovian case. In the short-time simulations, it is observed a significant difference between Markovian and non-Markovian cases, which can be applied to realize the environmental spectrum detection and enhance the measurement sensitivity in varying open quantum systems.

## Introduction

In real experiments, the dynamics of a quantum system can be significantly modified by the coupling with the surrounding environment^1–4^. So it is of great importance to study such problems in the framework of an open quantum system (OQS), $H_{tot}=H_{S}+H_{R}+H_{int}$, where $H_{S(R)}$ is the Hamiltonian of the central system (environment or reservoir), and $H_{int}$ characterizes the interaction Hamiltonian. The difficulties of solving the dynamics of an OQS usually arise from the large number of degrees of freedom of the reservoir^5,6^. Most often, the reduced density matrix of the central system is the key quantity to describe the interested central system, and is defined as the partial trace of the total system-reservoir density matrix over the degrees of freedom of the reservoir, $\rho _{S}(t) = \mathrm {Tr}_R[\rho _{tot}(t)]$. The evolution of the reduced density matrix $\rho _{S}$ is governed by master equations. In the past decades, several types of master equations have been developed to study different fields, e.g. Nakajima-Zwanzig equation^7,8^, Redfield master equation^9^, Lindblad master equation^10^, Hu-Paz-Zhang master equation^11^, general non-Markovian time-local master equation^12–16^, etc. With the master equation, the evolution of time-dependant expectation values for arbitrary operator in the Hilbert space of the system S, defined as $\langle O(t)\rangle = \mathrm {Tr}_{S\otimes R}[O\rho _{tot}(t)]$, can be obtained by solving a set of ordinary differential equations.

Of particular interest, two-time correlation functions are required for calculating the spectral function of the environment^17–24^. Clearly, master equations cannot directly derive the two- or multiple-time correlation functions. Only under the Born-Markov approximation, the two- or multi-time correlation functions can be calculated by the quantum regression theorem (QRT)^25–32^. Recent research indicates that we need to initiate our discussion in the non-Markovian regime, as the non-Markovian case is accurate and general. However, the QRT is no longer valid in the non-Markovian regime. In addition, due to the limited size of the reservoir, the energy exchange between system and environment can enforce the environment to approach non-equilibrium states, and consequently break the time translation invariance. Therefore, it is of importance to develop a systematic method to calculate the non-Markovian two-time correlation functions^33–40^.

A scheme to study the non-Markovian dynamics of OQS is called the stochastic Schrödinger equation (SSE) approach^41^, in which the influence from the environment on the state vector in the Hilbert space of the system is characterized by a Gaussian stochastic process. Every possible evolution of the state vector is a trajectory. The reduced density matrix for the system can be obtained numerically by taking the ensemble average over all possible trajectories. This process can also be conducted analytically and lead to a non-Markovian master equation^16,42^. In the Markovian case, the SSE and the corresponding master equation are in accordance with the results of Lindblad master equations. Therefore, it is natural to extend the SSE approach to calculate the two-time correlation functions (TTCF) or multi-time correlation functions (MTCF).

In the system-environment framework, the interaction Hamiltonian can be generally described by the Lindblad coupling operator *L*, and the total Hamiltonian is

1 $$\begin{aligned} H_{tot} = H_{S} + \lambda (L\sum _k g_kb_k^\dagger + L^\dagger \sum _k g_k^* b_k )+ H_R, \end{aligned}$$

where $H_{S(R)}$ is the Hamiltonian of the system (reservoir). $b_k$ and $b_k^\dagger$ are the annihilation and creation operators of the bosonic environment, $\lambda$ is a suitable perturbation parameter that can take one. $g_k (g_k^*)$ and $\omega _k$ are coupling constants and the eigen frequency of the *k*th mode of the environment, respectively.

In this paper, we derive a general formula for two-time correlation functions (TTCF) in the non-Markovian regime, where the TTCF is defined as

2 $$\begin{aligned} \langle A(t) B(t')\rangle = \mathrm {tr}_{S\otimes R}\left[ U^\dagger (t)AU(t)U^\dagger (t')BU(t')\rho _{tot}(0) \right] , \end{aligned}$$

where *A* and *B* are two arbitrary operators in the Hilbert space of the system, and $t>t'$ is always satisfied. Moreover, we assume that system and environment are decoupled at $t=0$, and the initial density matrix can be rewritten in the form of a pure state $\rho _{tot}(0) = |\psi _0\rangle \langle \psi _0|\otimes |0\rangle \langle 0|$, where $|\psi _0\rangle$ and $|0\rangle$ are the initial states of the system and the environment respectively. In an OQS, there are two major sources of noise, quantum fluctuations and thermal fluctuations. Recent experiments have successfully cooled the system down to the few milli-Kelvin regime and suppressed the thermal noises^43^. For simplicity, we study a zero-temperature environment and focus on the quantum fluctuations only.

The aim of the paper is twofold. Firstly, we derive a non-Markovian stochastic Schrödinger equation (SSE) to characterize the dynamics of TTCFs in accordance to the generalized system-environment Hamiltonian. The derived evolution functions for quantum mean values can be analytically governed by a single Gaussian noise. The subsequent process of numerical simulation for the ensemble average of all possible trajectories can be significantly simplified. Secondly, we investigate several typical applications of TTCF, e.g. two-level systems, and second-order correlation function $g^{(2)}$. In particular, we study how a TTCF is influenced by various non-Markovian environments, by adjusting the eigen frequencies of system and coupling strength. We discuss the physical mechanism in the model and the experimental feasibility of our proposed model.

## Background

In this section, we briefly review the stochastic Schrödinger equation (SSE) approach and compare it with the quantum regression theorem (QRT).

### Stochastic Schrödinger equation approach

The evolution of a general quantum open system (QOS), characterized by the Hamiltonian (1), is simply governed by the Schrödinger equation

3 $$\begin{aligned} \partial _t |\Psi (t)\rangle = -\frac{i}{\hbar } H_{tot}|\Psi (t)\rangle . \end{aligned}$$

Since the reservoir consists of a large number of bosonic oscillators, $H_R = \sum _k \omega _kb_k^\dagger b_k$, so it is natural to choose the Bargmann coherent state basis to represent the environment, $|z\rangle = \otimes _k|z_k\rangle$, a tensor product of all the environmental oscillators, where $|z_k\rangle$ is defined as $b_k|z\rangle = z_k|z_k\rangle$ for the kth mode in the environment. Therefore, the state vector of the system in the Bargmann basis, $|\psi _t(z^*) \rangle = \langle z|\Psi (t)\rangle$, can be expressed as a stochastic trajectory. In addition, its evolution, in the interaction picture, is governed by the following equation (setting $\hbar =1$)

4 $$\begin{aligned} \partial _t \langle z|\Psi (t)\rangle&= -i \langle z|H_{tot}|\Psi (t)\rangle \nonumber \\&= \left( -iH_{S} + \lambda Lz_t^* \right) \langle z|\Psi (t)\rangle -i \lambda L^\dagger \sum _k g_k^* e^{-i\omega _k t}\langle z|b_k|\Psi (t)\rangle , \end{aligned}$$

where the complex Gaussian noise $z_t^* = -i\sum _k g_k z_k^* e^{i\omega _kt}$, satisfying the auto-correlation function $\alpha (t,s)= \mathcal {M}[z_t^*z_s]$. The operation $\mathcal {M}[\cdot ] = \int d\mu (z) [\cdot ]$, where $d\mu (z)=\frac{d^2z e^{-|z|^2}}{\pi }$ and $\int d\mu (z)|z\rangle \langle z| = I$. Moreover, the evolution operator of system-environment *U*(*t*), defined as $|\Psi (t)\rangle = U(t)|\Psi (0)\rangle$, can be explicitly shown in the interaction picture $U(t) = e^{iH_R t} e^{-iH_{tot}t}$, which obeys the evolution equation

5 $$\begin{aligned} \partial _t U(t) = -i\left( H_S+ \lambda L\sum _k g_kb_k^\dagger e^{i\omega _k t}+ \lambda L^\dagger g_k^*b_k e^{-i\omega _k t} \right) U(t). \end{aligned}$$

For the cases that the system and environment are entangled initially, we can decompose the density matrix into a product state basis. Therefore, for simplicity, we assume the system and the environment are separate at $t=0$, and the environment is in the vacuum state, $|\Psi (0)\rangle = |\psi _0\rangle \otimes |0\rangle$. It is essential to calculate the term,

6 $$\begin{aligned} \langle z|b_k|\Psi (t)\rangle = \langle z|b_kU(t)|0\rangle |\psi _0\rangle . \end{aligned}$$

Here, we compare two equivalent methods, the Heisenberg equation approach and the quantum-state diffusion approach, to compute it. Firstly, let us consider the observable $b_k(t)= U^\dagger (t)b_k U(t)$ in the Heisenberg picture and its evolution equation acquires

7 $$\begin{aligned} \partial _t b_k(t) = U^\dagger (t) \left( -i\lambda g_k e^{i\omega _k t}L\right) U(t) = -i\lambda g_k e^{i\omega _k t}L(t). \end{aligned}$$

Inserting the formal solution of $b_k(t)$,

8 $$\begin{aligned} b_k(t)=b_k -i\lambda g_k \int _0^t ds e^{i\omega _k s}L(s), \end{aligned}$$

into the Eq. (6), we have

9 $$\begin{aligned} \langle z|U(t)b_k(t)|0\rangle = -i\lambda g_k \int _0^t ds\ e^{i\omega _k s}\langle z|U(t)L(s)|0\rangle . \end{aligned}$$

Consequently, the Eq. (4) is explicitly written in

10 $$\begin{aligned} \partial _t |\psi _t(z^*)\rangle = \left( -iH_{S} + \lambda Lz_t^* \right) |\psi _t(z^*)\rangle -\lambda ^2 L^\dagger \int _0^t ds \alpha (t,s) \langle z|U(t)L(s)|0\rangle |\psi _0\rangle , \end{aligned}$$

where the correlation function is $\alpha (t,s)=\sum _k |g_k|^2e^{-i\omega _k(t-s)}$ for the zero-temperature environment.

Secondly, we apply the chain rule of the functional derivative of the Gaussian stochastic process $z_t^*$ and the Eq. (6) can be turned as

11 $$\begin{aligned} \langle z| b_k U(t)|0\rangle = \frac{\partial }{\partial z_k^*} \langle z| U(t)|0\rangle = \int _0^t ds\ \frac{\partial z_s^*}{\partial z_k^*} \frac{\delta }{\delta z_s^*} \langle z|U(t)|0\rangle , \end{aligned}$$

and the consequent SSE is

12 $$\begin{aligned} \partial _t |\psi _t(z^*)\rangle = \left( -iH_S + \lambda Lz_t^* - \lambda ^2 L^\dagger \int _0^t ds \alpha (t,s)O(t,s,z^*) \right) |\psi _t(z^*)\rangle , \end{aligned}$$

where the *O* operator is the ansatz of the term $\frac{\delta }{\delta z_s^*}|\psi _t(z^*)\rangle = O(t,s,z^*)|\psi _t(z^*)\rangle$. In different models, the *O* operator can be resolved into the sum of a set of matrices with the undetermined coefficient functions. Due to the identity that $\frac{\partial }{\partial t} \frac{\delta }{\delta z_s^*} |\psi _t (z^*)\rangle = \frac{\delta }{\delta z_s^*} \frac{\partial }{\partial t}|\psi _t (z^*)\rangle$, the *O* operator is governed by a nonlinear evolution function

13 $$\begin{aligned} \partial _t O(t,s,z^*) = \left[ -iH_S+\lambda Lz_t^* -\lambda ^2L^\dagger \bar{O}(t,z^*),\ O(t,s,z^*) \right] - \lambda L^\dagger \frac{\delta \bar{O}(t)}{\delta z_s^*}, \end{aligned}$$

where the $\bar{O}$ operator is defined as $\bar{O}(t,z^*) = \int _0^t ds \alpha (t,s)O(t,s,z^*)$. Once the matrices basis of the *O* operator is determined, all undetermined coefficients can be numerically simulated.

By comparing the results of Eqs. (10) and (12), the initial condition of *O* operator must satisfy

14 $$\begin{aligned} O(t,s=t,z^*)|\psi _t(z^*)\rangle = L |\psi _t(z^*)\rangle . \end{aligned}$$

SSE approach can be used to solve many OQS models, including discrete qubit systems, continuous variable systems, and multi-layer mixes systems in the presence of non-Markovian environments^44,45^. It can also be extended to deal with both zero-temperature and finite temperature environments.

### Quantum regression theorem

Quantum regression theorem indicates that one-time expectation values of system operators and TTCFs (MTCFs) have the same form of evolution equations^25–27^. To compute the expectation values, we introduce the Liouville operator with respect to time, ${\mathcal {L}}(t)$ and get the reduced density matrix as

15 $$\begin{aligned} \partial _{t} \rho _S(t) = {\mathcal {L}}(t) \rho _S(t). \end{aligned}$$

It is important to mention that this is assuming that there are no non-linear dynamics involved in the environment. From this equation, we can find the mean value of a system observable *A* is determined by

16 $$\begin{aligned} \partial _{t}\langle A(t) \rangle = \mathrm {tr}_S[ A (0) {\mathcal {L}}(t) \rho _S(t) ]. \end{aligned}$$

For the two-time correlation functions, we keep the first operator as *A*(*t*) and the second operator is denoted as $B(t')$. Assuming the time $t'$ is fixed and $t>t'$, the TTCF is expressed as

17 $$\begin{aligned} \langle A(t)B(t')\rangle&= \mathrm {tr}_{S}[A\rho _{tot,B}(t',\tau )], \end{aligned}$$

where $\rho _{tot,B}(t',\tau ) = \mathrm {tr}_R[B(\tau )\rho _{tot}(t'+\tau )]$, and the time index $\tau =t-t'$. Under the Born-Markov approximation, $\rho _{tot}(t)$ is always given by $\rho _S(t)\otimes \rho _R(0)$, and consequently, $\rho _{tot,B}(t',\tau ) = B\rho _S(t'+\tau )$. It indicates that, for a given $t'$, the new reduced density matrix $\rho _{tot,B}$ evolves in the similar manner as $\rho _S$,

18 $$\begin{aligned} \partial _{\tau }\rho _{tot,B} = \mathcal {L}(\tau )\rho _{tot,B}. \end{aligned}$$

Therefore, the TTCF is downgraded to a single operator mean value, that

19 $$\begin{aligned} \partial _{\tau } \langle A(t'+\tau )B(t')\rangle =\mathrm {tr}_S[ A \mathcal {L}(\tau )\rho _{tot,B}]. \end{aligned}$$

The SSE approach can derive the same result. Formally, the reduced density matrix of the system can be obtained by taking ensemble average over all possible trajectories, either analytically or numerically, $\rho _S(t) = \mathcal {M}[|\psi _t(z^*)\rangle \langle \psi _t(z)|]$. Consequently, the formal master equation can be expressed as

20 $$\begin{aligned} \partial _t \rho _{S} = -i[H_{S},\,\rho _{S}] +[L,\,\mathcal {M}(|\psi _t\rangle \langle \psi _t|\bar{O}^\dagger )] - [L^\dagger ,\,\mathcal {M}(\bar{O}|\psi _t\rangle \langle \psi _t|)]. \end{aligned}$$

If the environment is Markovian and the correlation function, $\alpha (t,s)=\frac{\Gamma }{2}\delta (t,s)$, the formal master equation can be expressed in the Lindblad form (the temperature is zero, for simplicity):

21 $$\begin{aligned} \partial _t\rho _S=-i[H_{S},\,\rho _{S}] +\frac{\Gamma }{2}\left( 2L\rho _S L^\dagger - L^\dagger L\rho _S -\rho _SL^\dagger L \right) . \end{aligned}$$

Therefore, the evolution equation of one-time expectation values of system operators is

22 $$\begin{aligned} \partial _t \langle A(t)\rangle&= \mathrm {tr}_S(A \partial _t\rho _S(t)) \nonumber \\&= -i\langle [A,\ H_S] \rangle +\frac{\Gamma }{2}(\langle 2L^\dagger AL\rangle - \langle L^\dagger L A \rangle - \langle A L^\dagger L\rangle ). \end{aligned}$$

For TTCFs, assuming the time $t'$ is fixed and $t>t'$, the evolution equation is expressed

23 $$\begin{aligned} \partial _t\langle A(t)B(t')\rangle&= \partial _t\mathrm {tr}_{S\otimes R} (A \ U(\tau )B\rho _{tot}(t')U^\dagger (\tau ))\nonumber \\&= \mathrm {tr}_S\left( A\ \partial _{\tau }\mathcal {P}_S(\tau ) \right) , \end{aligned}$$

where $\mathcal {P}_S(\tau )=\mathrm {tr}_R(P_S(\tau ))=\mathrm {tr}_R( U(\tau )B\rho _{tot}(t')U^\dagger (\tau ))$, and $\tau = t-t'$. Following the similar method, the evolution equation of the term $\mathcal {P}_S(\tau )$ is

24 $$\begin{aligned} \partial _{\tau } \mathcal {P}_S(\tau ) = -i[H_{S},\, \mathcal {P}_S]+[L,\,\mathcal {M}(P_S\bar{O}^\dagger )] - [L^\dagger ,\,\mathcal {M}(\bar{O}P_S)]. \end{aligned}$$

In the Markoivan regime, it is easy to show that the term $\mathcal {P}_S(t)$ follows the same operator equation form as the master equation,

25 $$\begin{aligned} \partial _{\tau } \mathcal {P}_S(\tau ) = -i[H_{S},\,\mathcal {P}_S] +\frac{\Gamma }{2}\left( 2L\mathcal {P}_S L^\dagger - L^\dagger L\mathcal {P}_S -\mathcal {P}_SL^\dagger L \right) . \end{aligned}$$

Consequently, we can conclude that the TTCF evolution equation can be expressed as the same as the single-time expectation value evolution equation

26 $$\begin{aligned} \partial _t \langle A(t)B(t')\rangle = -i\langle [A(t)B(t'),\ H_S] \rangle +\frac{\Gamma }{2}(\langle 2L^\dagger A(t)B(t')L\rangle - \langle L^\dagger L A(t)B(t') \rangle - \langle A(t)B(t') L^\dagger L\rangle ). \end{aligned}$$

However, from our derivations, it is clear to show that the QRT is not valid in general conditions . Particularly, when the environment is structured where the Markovian assumption is no longer applicable, the evolution equation of TTCFs needs to compute in the language of non-Markovian OQS carefully. In some previous work, the SSE approach had been applied to compute the stochastic propagator where two independent stochastic processes are employed. From our experience, the convergence speed usually depends on the type of system-environment coupling and the size of the system’s Hilbert space. The operation that computes the ensemble average over multiple stochastic processes will increase the computational burden significantly. To solve this issue, our approach can generally calculate TTCFs with only applying one stochastic noise.

## Method

We consider a general OQS coupled to a bosonic environment with a general Hamiltonian (1) in the interaction picture,

27 $$\begin{aligned} H_{tot} = H_{S} + \lambda L\sum _k g_k b_k^\dagger e^{i\omega _k t} + \lambda L^\dagger \sum _k g_k^* b_k e^{-i\omega _k t}, \end{aligned}$$

and the corresponding SSE of the system’s state vector, $|\psi _t(z^*)\rangle = \langle z|\Psi (t)\rangle$, is

28 $$\begin{aligned} \partial _t |\psi _t(z^*)\rangle&= \left( -iH_S + \lambda Lz_t^*\ - \lambda L^\dagger \int _0^t ds \alpha (t,s) \frac{\delta }{\delta z_s^*}\right) |\psi _t(z^*)\rangle . \end{aligned}$$

The evolution functions of the TTCFs are formally expressed as

29 $$\begin{aligned} \partial _{t} \langle A(t) B(t')\rangle&= \partial _{t}\mathrm {tr}_{S\otimes R}\left[ U^\dagger (t)AU(t)U^\dagger (t')BU(t')\rho _{tot}(0) \right] \nonumber \\&= \mathrm {tr}_S\left[ A \int d\mu (z)\partial _{t} \langle z| U(t)U^\dagger (t')BU(t') |\psi _0,0\rangle \langle \psi _0,0|U^\dagger (t) |z\rangle \right] . \end{aligned}$$

Here, we need to point out that $U(t)U^\dagger (t') \ne U(t-t')$ in the interaction picture. And we define the two terms

$$\begin{aligned} |\eta (t,t',z^*) \rangle&= \langle z| U(t)U^\dagger (t')BU(t') |\psi _0,0\rangle , \\ |\psi _{t}(z)\rangle&= \langle z|U(t) |\psi _0,0 \rangle , \end{aligned}$$

which can be simulated independently with the same set of stochastic noise $z_t^*$. To contact with the previous work, particularly comparing with the method in the ref.^18^, we insert the identity of a second independent noise $w_t^*$ into $|\eta (t_1,t_2,z^*)\rangle$ and turn it into

30 $$\begin{aligned} |\eta (t,t',z^*)\rangle&= \int d\mu (w) \langle z| U(t)U^\dagger (t')|w\rangle B |\psi _{t'}(w^*)\rangle , \end{aligned}$$

where $d\mu (w) = d^2w e^{-|w|^2} / \pi$ and the term $\langle z| U(t)U^\dagger (t')|w\rangle$ is a stochastic propagator. Once being expanded into two independent noise-basis, every element of the propagator can be independently characterized as a stochastic function of noises $z_t^*$ and $w_t^*$. However, the computation complexity is also doubled. And the consequent TTCF (29) evolves

31 $$\begin{aligned} \partial _{t} \langle A(t) B(t')\rangle&= \mathrm {tr}_S\left[ A \int d\mu (z)\int d\mu (w) \partial _{t} \langle z| U(t)U^\dagger (t')|w\rangle B|\psi _{t'}(w^*)\rangle \langle \psi _{t}(z)| \right] . \end{aligned}$$

For those simple OQSs, e.g. consisting of few qubits, this approach is helpful in calculating TTCFs and MTCFs. Our approach will only employ one stochastic noise $z_t^*$ to simulate the TTCF, which can be easily extended to a complicate-structured quantum model. Applying the similar derivation in the Eq. (12), we arrive at the time-convolutionless evolution equation of $\eta (t,t',z^*)\rangle$,

32 $$\begin{aligned} \partial _{t}|\eta (t,t',z^*)\rangle&= \left( -iH_S +\lambda Lz_t^* \right) |\eta (t,t',z^*)\rangle -i\lambda L^\dagger \sum _k g_k^* e^{-i\omega _k t}\langle z|b_k U(t) U^\dagger (t')BU(t')|\psi _0,0\rangle \nonumber \\&=\left( -iH_S +\lambda Lz_t^* -\lambda L^\dagger \int _0^t ds\ \alpha (t,s) \frac{\delta }{\delta z_s^*}\right) |\eta (t,t',z^*)\rangle , \end{aligned}$$

where the functional deferential chain rule in Eq. (11) is applied again. Compute the last term of the Eq. (32),

33 $$- iL^{\dag } \sum\limits_{k} {g_{k}^{*} } e^{{ - i\omega _{k} t}} \langle z|b_{k} U(t)U^{\dag } (t^{\prime})BU(t^{\prime})|\psi _{0} ,0\rangle = - iL^{\dag } \sum\limits_{k} {g_{k}^{*} } e^{{ - i\omega _{k} t}} \langle z|U(t)b_{k} U^{\dag } (t^{\prime})BU(t^{\prime})|\psi _{0} ,0\rangle - \lambda L^{\dag } \int_{0}^{t} d s\alpha (t,s)\langle z|U(t)L(s)U^{\dag } (t^{\prime})BU(t^{\prime})|\psi _{0} ,0\rangle.$$

In the first term of the above expression, it should be noted that $\tilde{b_k}(t) = U(t)b_kU^\dagger (t)$ cannot apply the analytical solution of $b_k(t)$ in Eq. (8) directly. In order to apply it, we need to slightly adjust the form of $\tilde{b_k}(t)$ to

34 $$\begin{aligned} \tilde{b_k}(t)&= e^{iH_R t} U^\dagger (-t)b_k e^{i\omega _k t} U(-t)e^{-iH_R t} \nonumber \\&= e^{iH_R t} e^{i\omega _k t} \left( b_k - i\lambda g_k\int _0^{-t}\ ds e^{i\omega _k s} L(s)\right) e^{-iH_R t}, \end{aligned}$$

where the term $e^{i\omega _k (t+s)}$ is zero, due to the rotating wave approximation. Consequently, the above expression can be simplified as

35 $$\begin{aligned} \tilde{b_k}(t) = e^{iH_R t} e^{i\omega _k t} \left( b_k \right) e^{-iH_R t} = b_k. \end{aligned}$$

After repeating the above approach, we can obtain the complete form of the evolution equation of the term $|\eta (t,t',z^*)\rangle$,

36 $$\partial _{t} |\eta (t,t^{\prime},z^{*} )\rangle = \left( { - iH_{S} + \lambda Lz_{t}^{*} } \right)|\eta (t,t^{\prime},z^{*} )\rangle - \lambda ^{2} L^{\dag } \int_{0}^{t} d s\alpha (t,s)\langle z|U(t)L(s)U^{\dag } (t^{\prime})BU(t^{\prime})|\psi _{0} ,0\rangle {\text{ }} - \lambda ^{2} L^{\dag } \int_{0}^{{t^{\prime}}} d s\alpha (t,s)\langle z|U(t)U^{\dag } (t^{\prime})BU(t^{\prime})L(s)|\psi _{0} ,0\rangle.$$

### Short-time limit

In the short time-scale, a regime where non-Markovian effects are dominant, we consider the $t'=0$ condition and the subsequent TTCF is simplified and expressed as

37 $$\begin{aligned} \langle A(t) B(0)\rangle = \mathrm {tr}_{S\otimes R}\left[ U^\dagger (t)AU(t)B\rho _{tot}(0) \right] . \end{aligned}$$

In the following derivation, set the parameter $\lambda =1$ for simplicity. In accordance with the short-time assumption, the evolution equation of $|\eta \rangle$ in the Eq. (36) can be further simplified as

38 $$\begin{aligned} \partial _t |\eta (t,z^*)\rangle&= \left( -iH_S +Lz_t^* \right) |\eta (t,z^*) \rangle -L^\dagger \int _0^t ds \alpha (t,s)\langle z|U(t)L(s)B|\psi _0,0\rangle . \end{aligned}$$

We notice that the evolution equation of $|\eta (t,z^*)\rangle$ has the same operator form as $|\psi _t(z^*)\rangle$, in the Eq. (10). In addition, the evolution equation can be alternatively written in the form of SSE, with the ansatz *O* operator. Comparing the two equivalent approaches, we find out that

39 $$\begin{aligned} \partial _t |\eta (t,z^*)\rangle = \left( -iH_S +Lz_t^* -L^\dagger \int _0^t ds O(t,s,z^*)\right) |\eta (t,z^*)\rangle , \end{aligned}$$

where the anstz $O(t,s,z^*)$ operator is the same as the *O* operator used in Eq. (12), with the same initial value $O(t,s=t)=L$ and the same operator form evolution equation (13). But $|\eta (t,z^*)\rangle$ and $|\psi _t(z^*)\rangle$ still characterize two different sets of trajectories, due to the different initial values of $|\eta (t=0,z^*)\rangle = B|\psi _0\rangle$ and $|\psi _0\rangle$ respectively. Now, we can conclude that the TTCF in the short-time limit can be computed by taking the ensemble average over two trajectories

40 $$\begin{aligned} \langle A(t)B\rangle = \mathrm {tr}_S[A \mathcal {M}(R(t))], \end{aligned}$$

where $R(t)=|\eta (t,z^*)\rangle \langle \psi _t(z)|$ is the equivalent stochastic density matrix. Similar to the master equation approach, $\mathcal {M}(R(t))$ can be determined by an evolution equation

41 $$\begin{aligned} \partial _t \mathcal {M}(R(t))= [-iH_S,\ \mathcal {M}(R(t))] + [L, \ \mathcal {M}(R(t)\bar{O}^\dagger )]-[L^\dagger , \ \mathcal {M}(\bar{O}R(t))]. \end{aligned}$$

Here, the subsequent evolution equation of the short-time limit TTCF is

42 $$\begin{aligned} \partial _t \langle A(t)B\rangle&= \mathrm {tr}_S[A\partial _t \mathcal {M}(R(t))] \nonumber \\&= \mathrm {tr}_S\left[ A [-iH_S,\ \mathcal {M}(R(t))] + A[L, \ \mathcal {M}(R(t)\bar{O}^\dagger )]-A[L^\dagger , \ \mathcal {M}(\bar{O}R(t))] \right] . \end{aligned}$$

Generally, the *O* operator can be resolved in a sum of operator basis with the ascending order of noises. From the solution, we can prove that QRT is not valid in a general OQS.

However, in the Markovian limit, the *O* operator equals to the Lindblad operator *L*, and the correlation function $\alpha (t,s) = \frac{\Gamma }{2}\delta (t,s)$. Consequently, the operator $\mathcal {M}(R(t)\bar{O}^\dagger )$ can be explicitly expressed as $\frac{\Gamma }{2}\mathcal {M}(R(t))L^\dagger$. The subsequent evolution equation of TTCF can be further simplified to

43 $$\begin{aligned} \partial _t\langle A(t)B\rangle&= \mathrm {tr}_S\left[ A [-iH_S,\ \mathcal {M}(R(t))] + \frac{\Gamma }{2}A([L, \ \mathcal {M}(R(t)L^\dagger ]-[L^\dagger , \ L\mathcal {M}(R(t))] \right] \nonumber \\&= \mathrm {tr}_S[\left( [iH_s,\ A] +\frac{\Gamma }{2} (L^\dagger AL - [A,\ L^\dagger L]_+)\right) \mathcal {M}(R(t))], \end{aligned}$$

where $[A,\ B]_+ = AB+BA$ is the anticommutator. It is clear that the QRT is not valid even the environment is Markovian.

### General discussion

In order to study the genreal TTCF of OQSs in the non-Markovian regime, we derive the general formula of TTCFs from Eq. (32) by expanding the $|\eta (t,t',z^*)\rangle$ in a linear sum of ascending order of noise terms

44 $$\begin{aligned} |\eta (t,t',z^*)\rangle = |\eta _0(t,t')\rangle +\sum _k \int _0^t \frac{ds_1...ds_k}{k!} (\prod _k z^*_{s_k}) |\eta _k(t,t',s_1,..,s_k)\rangle . \end{aligned}$$

By substituting the expansion into Eq. (32), we can equal the terms in two sides of the equation with the same order of noise, and obtain a series of deferential equations for each noise-independent $|\eta _k\rangle$,

45 $$\begin{aligned} \partial _t |\eta _k\rangle = -iH_s|\eta _k\rangle - \lambda L^\dagger \int _0^t \alpha (t,s)|\eta _{k+1}\rangle . \end{aligned}$$

In addition, we notice the terms containing $z_t^*$ in the two sides of the Eq. (32) are always equal to each other, so that the initial conditions of $|\eta _k\rangle$ can be obtained

46 $$\begin{aligned} \eta _k(t,t',s=t,s_1,..,s_{k-1}) = L\eta _{k-1}(t,t',s_1,..,s_{k-1}). \end{aligned}$$

Up to now, we apply the SSE approach and employ only one noise to calculate the non-Markovian TTCFs by generating evolution equations. This method starts from the microscopic OQS Hamiltonian, and is tolerant to many factors, including the central system Hamiltonian, the system-environment coupling operator, the correlation functions of the non-Markovian environment, etc. So it can be used to systematically solve TTCFs of OQS.

## Numerical results

To examine the derived TTCF equations, we consider a dissipative two-level system $H_s = \frac{\omega }{2}\sigma _z$, in which the dissipative type of coupling is $L=\sigma _-$. In refs.^13,14,46^, the *O* operator has been discussed in detail, that $O(t,)=f(t,s)\sigma _-$, where the coefficient factor *f*(*t*, *s*) is governed by the initial condition $f(t,s=t)=1$ and the evolution function

47 $$\begin{aligned} \partial _t f(t,s) = i\omega f+ F(t)f(t,s), \end{aligned}$$

where $F(t)= \int _0^t\ d\tau \alpha (t,\tau )f(t,\tau )$ and $\alpha (t,s)$ is the correlation function. The subsequent SSE and master equation are explicitly expressed

48 $$\begin{aligned} \partial _t |\psi _t(z^*)\rangle = \left( -iH_s +Lz_t^* -F L^\dagger L \right) |\psi _t(z^*)\rangle ,\nonumber \\ \partial _t \rho = [-iH_s,\ \rho ]+F^*[L,\ \rho L^\dagger ] - F[L^\dagger , \ L\rho ]. \end{aligned}$$

We now calculate the non-Markovian evolution equations of TTCFs, by applying the commutation relations between the Pauli matrices. First, we consider the evolution of the mean averages $\langle \sigma _i \rangle , i=(x, y, z, +, -)$ from the derived master Eq. (48), which results in a group of linear differential equations:

$$\begin{aligned} \partial _t \langle \sigma _x \rangle&= -\omega \langle \sigma _y\rangle -F^*\langle \sigma _+\rangle -F \langle \sigma _-\rangle , \\ \partial _t \langle \sigma _y \rangle&=\omega \langle \sigma _x\rangle +iF^*\langle \sigma _+\rangle -iF \langle \sigma _-\rangle ,\\ \partial _t \langle \sigma _z \rangle&= 0,\\ \partial _t\langle \sigma _+ \rangle&= i\omega \langle \sigma _+ \rangle -F^*\langle \sigma _+ \rangle ,\\ \partial _t\langle \sigma _- \rangle&= -i\omega \langle \sigma _- \rangle -F\langle \sigma _- \rangle , \end{aligned}$$

Then, we calculate the TTCFs by applying the results of short-time limit (42) and general derivation respectively and compare the difference.

### Case I: short-time limit

In the short-time limit, set the time index $t'=0$, and TTCFs can be obtained by the following set of differential equations:

$$\begin{aligned} \partial _t \langle \sigma _x(t)\sigma _y \rangle&= \mathrm {tr}_S\left[ \sigma _x [-i\frac{\omega }{2}\sigma _z,\ \mathcal {M}(R(t))] + \sigma _x[\sigma _-, \ \mathcal {M}(R(t))F^*\sigma _+]-\sigma _x[\sigma _+, \ F\sigma _-\mathcal {M}(R(t))] \right] \\&= \langle \left( [\frac{i\omega }{2}\sigma _z,\ \sigma _x] + (F+F^*)\sigma _+\sigma _x\sigma _- - F \sigma _x\sigma _+\sigma _- -F^* \sigma _+\sigma _-\sigma _x\right) (t)\sigma _y\rangle \\&=-\omega \langle \sigma _y(t)\sigma _y\rangle -F^*\langle \sigma _+(t)\sigma _y\rangle -F \langle \sigma _-(t)\sigma _y\rangle , \end{aligned}$$

and similarly,

49 $$\begin{aligned} \partial _t \langle \sigma _y(t)\sigma _y \rangle&= \omega \langle \sigma _x(t)\sigma _y \rangle +iF^*\langle \sigma _+(t)\sigma _y \rangle -iF\langle \sigma _-(t)\sigma _y \rangle ,\nonumber \\ \partial _t \langle \sigma _+(t)\sigma _y \rangle&= i\omega \langle \sigma _+(t)\sigma _y \rangle - F^*\langle \sigma _+(t)\sigma _y \rangle , \nonumber \\ \partial _t \langle \sigma _-(t)\sigma _y \rangle&= -i\omega \langle \sigma _-(t)\sigma _y \rangle -F\langle \sigma _-(t)\sigma _y \rangle . \end{aligned}$$

**Figure 1:**
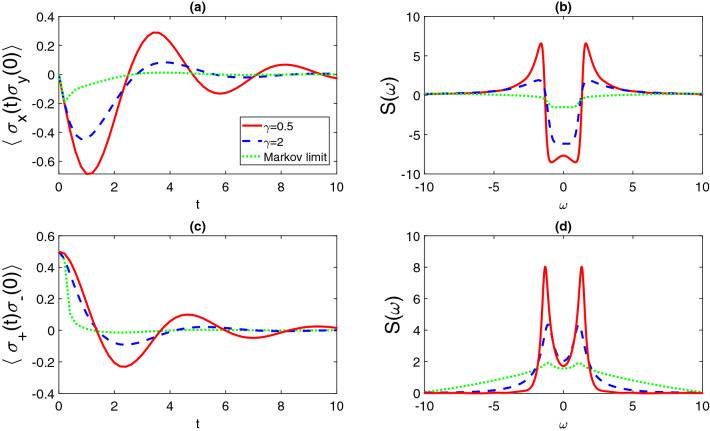
Dynamics of two-time correlation functions in the short-time limit for (**a**) $\langle \sigma _x(t)\sigma _y(0)\rangle$ and (**c**) $\langle \sigma _+(t)\sigma _-(0)\rangle$, in the Markov limit (green dotted line, $\gamma =10$), and the non-Markovian regime ($\gamma =0.5$ red solid line, and $\gamma =2$ blue dashed line). The Fourier spectral functions, $\mathrm {S}(\omega )$, of $\langle \sigma _x(t)\sigma _y(0)\rangle$ and $\langle \sigma _+(t)\sigma _-(0)\rangle$ are shown in (**b**,**d**) respectively. Other parameters are chosen as $\omega =1$, $\Gamma =1$.

In order to study the impacts on TTCFs induced by the non-Markovian environment, we use the Ornstein-Uhlenbeck style correlation function,

50 $$\begin{aligned} \alpha (t,s) = \frac{\Gamma \gamma }{2}e^{-\gamma |t-s|}, \end{aligned}$$

where $\Gamma$ is the global coupling strength and the factor $\gamma$ indicates the spectral width of the coupling. In addition, $1/\gamma$ scales the memory time of the environment. Therefore, if $\gamma$ takes infinity, the environment does not have memory effect and the system-environment interaction is close to the Markov limit. Note that our method works for arbitrary spectral functions and the corresponding correlation functions of the environment, including Ohmic, super- and sub-Ohmic, etc. Without loss of generality, the initial state of the system is chosen to be $|\psi _0\rangle = (|0\rangle + |1\rangle )/\sqrt{2}$. In Fig. 1, we select $\gamma =0.5$ and 2 to simulate the non-Markovian dynamics and compare with the Markovian limit when $\gamma =10$. Two TTCFs, $\langle \sigma _x(t)\sigma _y(0)\rangle$ and $\langle \sigma _+(t)\sigma _-(0)\rangle$, are investigated as the example. We observe considerable difference between the non-Markovian and Markovian dynamics that the non-Markovian dynamics oscillate deeper and last longer time to achieve the steady value, while the Markov dynamics quickly decay to its long-time limit value. In addition, from the microscopic perspective, the Markovian environment indicates the equal interaction strength to every mode, a flat spectral function. This can be observed in Fig. 1b and (d), that the green dotted line is very close to a flat line when the coupling is Markovian. Since the selected Ornstein-Uhlenbeck correlation function can gradually show the transition from Markov to non-Markovian regime, we witness the peaks of the Fourier spectrum are higher and sharper at the resonant location $\pm \omega$. It indicates the strong non-Markovian effects occur in the short-time regime. Moreover, some sub-peaks of the spectrum can only be observed in the non-Markovian regime.

### Case II: general TTCFs

Here, we will continue to discuss the TTCF in a more general manner, where the $t'$ takes a non-zero value. By expanding the noisy term $|\eta \rangle$ into a polynomial consisting of ascending orders of noise $z_{s_k}^*$, as shown in Eq. (45), our method can be applied to systematically solve many models. For this two-level dissipative system, we have proved that the order of noise in the trajectory will not exceed 1 in our previous study. With the exact $|\eta _0\rangle$ and $|\eta _1\rangle$, governed by

$$\begin{aligned} \partial _t \eta _0&= -iH_s\eta _0 - L^\dagger \int _0^t ds\ \alpha (t,s) \eta _1\\ \partial _t \eta _1&= -iH_s \eta _1 , \end{aligned}$$

and the boundary condition

51 $$\begin{aligned} \eta _1(t,t', s=t) = L\eta _0(t,t'), \end{aligned}$$

the noisy trajectory $|\eta (t,t',z^*)\rangle$ can be simulated as

52 $$\begin{aligned} |\eta (t,t',z^*)\rangle = |\eta _0\rangle + \int _0^t ds_1\ z_{s_1}^*|\eta _1\rangle . \end{aligned}$$

When $t=t'$, the initial condition is $|\eta \rangle = B|\psi _t(z^*)\rangle$. Then we can solve the TTCF $\langle A(t)B(t')\rangle$ numerically stating from arbitrary $t'$. Here, we take the TTCF $\langle \sigma _x(t)\sigma _z(t')\rangle$ as an example.

**Figure 2:**
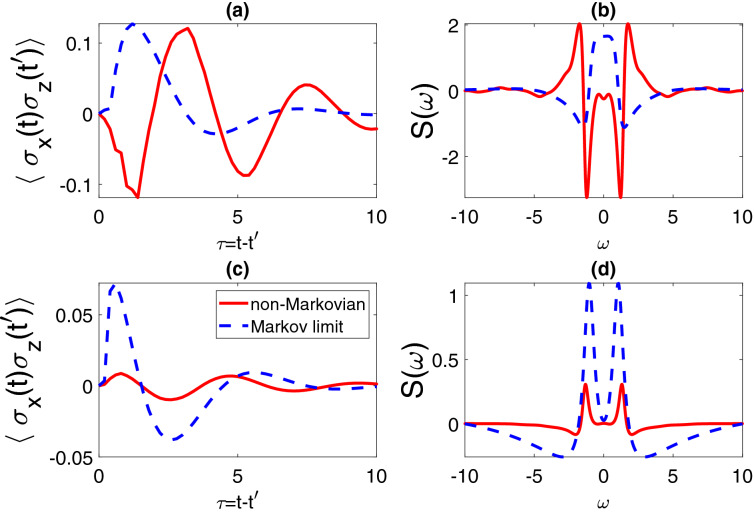
Dynamics of two-time correlation functions in the short-time limit for $\langle \sigma _x(t)\sigma _z(t')\rangle$, with two initial conditions: (**a**) $t'=5$ and (**c**) $t'=10$, in the Markov limit (blue dashed line), and non-Markovian regime (red solid line). The corresponding Fourier spectral functions are shown in (**b**,**d**) respectively. Other parameters are chosen as $\omega =1$, $\Gamma =1$.

In Fig. 2a, we display the evolution of $\langle \sigma _x(t)\sigma _z(t')\rangle$ as the function of the time difference $\tau =t-t'$, and choose $t'=5$. In Fig. 2c, the $t'$ is changed to 10. The Fourier spectral functions of the two TTCFs are shown in Fig. 2b,d respectively. We also compare the dynamics in the Markovian (blue dashed line) and non-Markovian (red solid line) regimes. In the non-Markovian regime, there is a significant difference between the two TTCFs: $\langle \sigma _x(\tau +5)\sigma _z(5)\rangle$ (Fig. 2a) and $\langle \sigma _x(\tau +10)\sigma _z(10)\rangle$ (Fig. 2c), which indicates that the QRT and the time translation invariance are not valid. Particularly, in Fig. 2a, we observe that the dynamics does not change gradually with the change of $\gamma$, but a sudden change. In contrast, the two Markovian dynamics in (a) and (c) are similar to each other, which indicates that the QRT can be valid with the Born-Markov approximation. Moreover, the absolute magnitude of $\langle \sigma _x(\tau +10)\sigma _z(10)\rangle$ in (c) is much smaller, comparing to the value in (a). It is because the dissipative system loses energy into the environment gradually and the correlation for the long time difference vanishes quickly. Therefore, the short-time approximation is a reasonable way to simplify the problem. However, for some other complicated models, such as the spin-boson model, the TTCF will show significant differences even in the long-time limit.

## Conclusion

In this paper, we have derived the exact evolution equation of the non-Markovian two-time correlation function for general open quantum system models, using the stochastic Schödinger equation approach. This method allows us to turn the hard open quantum system problems into a numerical ensemble average problem over all possible trajectories. Starting from a fully microscopic Hamiltonian, we can obtain a time-local SSE for the trajectory. In addition, our method employs one Gaussian noise to simulate two different trajectories and reduces the computational complexity significantly, which makes it possible to solve complicated and large-size models. Moreover, our method can be extended to study the multiple time correlation functions. Our numerical results disclose a fact that the non-Markovian TTCF is far different from the Markov limit, especially when the time is short, a strong non-Markovian regime. Our work can help better understand the dynamics of system in the presence of a non-equilibrium environment in a full quantum framework, and extend the research of non-Markovian quantum effects to more complicated chemical and biological systems.
